# Age of Onset Moderates the Association between Total Antioxidant Capacity and Cognitive Deficits in Patients with Drug-Naïve Schizophrenia

**DOI:** 10.3390/antiox12061259

**Published:** 2023-06-12

**Authors:** Jiaxin Li, Deyang Li, Junru Guo, Dongmei Wang, Xiangyang Zhang

**Affiliations:** 1 CAS Key Laboratory of Mental Health, Institute of Psychology, Chinese Academy of Sciences, 16 Lincui Road, Chaoyang District, Beijing 100101, China; 2 Department of Psychology, University of Chinese Academy of Sciences, Beijing 100049, China; 3 Department of Psychology, Guizhou Minzu University, Guiyang 550025, China

**Keywords:** schizophrenia, age of onset, TAOC, cognitive function, oxidative stress

## Abstract

Schizophrenia patients with an earlier age of onset have been found to have more serious negative symptoms and cognitive deficits. Oxidative stress is thought to be implicated in cognitive impairment in schizophrenia. Total antioxidant capacity (TAOC) is an essential indicator of oxidative stress. However, the association between age of onset, TAOC, and cognitive performance in schizophrenia remains unexplored. In this study, 201 patients (age: 26.5 ± 9.6 years; male: 53.2%) with drug-naïve schizophrenia were recruited. Clinical symptoms were evaluated using the Positive and Negative Syndrome Scale (PANSS). Cognitive functioning was assessed using the Repeatable Battery for the Assessment of Neuropsychological Status (RBANS). Plasma TAOC levels were analyzed using established procedures. Results showed that early-onset (EO) patients had higher TAOC levels, more severe negative symptoms and performed worse on visuospatial/constructional, language and RBANS total scores than non-EO patients. After Bonferroni correction, only non-EO patients showed a significant inverse relationship between TAOC levels and RBANS language, attention, and total scores. Our findings suggest that an early/late age of onset may be correlated with psychopathological symptoms, cognitive impairment and oxidative responses in schizophrenia. Furthermore, the age of onset may moderate the relationship between TAOC and cognitive function in patients with schizophrenia. These findings suggest that improving oxidative stress status in non-EO schizophrenia patients may enhance their cognitive function.

## 1. Introduction

Schizophrenia is one of the psychiatric disorders covering a broad range of psychiatric symptoms, with an estimated global prevalence of 0.28% [1]. In China, the lifetime prevalence of schizophrenia is estimated to be 0.54% [2], with a prevalence of disability of 0.41% [3]. Individuals with schizophrenia typically exhibit disorganized formal thoughts, delusions, hallucinations, catatonic symptoms, affective disorders, and neurocognitive deficits [4]. Schizophrenia is a diverse disorder, and one aspect of its diversity is the age of onset. Because the presentation of schizophrenia can vary significantly across ages, the age of onset is generally used to anticipate schizophrenia outcomes [5]. A common definition of early-onset (EO) schizophrenia is that the patient develops psychotic symptoms before age 21 [6]. Early-onset schizophrenia patients were shown to have more clinically severe symptoms and more impaired cognitive performance [7]. Mechanisms for these associations may involve neurotrophic factors [8], oxidative stress [9,10], differences in neurodevelopment [11], and differences in lifestyle and dietary patterns between ages [12].

Schizophrenia is associated with cognitive impairments such as deficits in working memory, attention, and visual and verbal learning [13]. Around 98% of schizophrenia patients experience cognitive impairments, which severely and negatively impact the patient’s overall functioning and hinder recovery [14]. As the three main characteristics of schizophrenia, positive symptoms, negative symptoms, and cognitive deficits are closely correlated [15]. Studies have shown that the severity of negative and cognitive symptoms is associated with impairments in semantic memory, verbal memory, and executive functioning, whereas positive symptoms are associated with semantic memory [16]. Schizophrenia usually develops during adolescence or young adulthood, when the most dramatic cognitive decline may be observed [14]. In contrast, cognitive function is better preserved in patients with late-onset schizophrenia [17]. Several studies have found that an earlier age of onset in schizophrenia is related to more positive symptoms, negative symptoms, poorer immediate memory, attention, social cognition, and verbal learning functions [18,19,20].

Disturbances in the oxidative stress system are suggested to influence the etiology and cognitive impairment of schizophrenia [21]. One study suggested that in early-onset schizophrenia patients, glutathione levels are decreased, and lipid peroxidation (LOOH) levels are higher, indicating that disturbances in the oxidative stress system might impact the earlier age of onset of schizophrenia [22]. Another study found that superoxide dismutase (SOD) activity is positively correlated with the severity of negative symptoms and inversely associated with the performance of cognitive function tests in late-life schizophrenia [23]. Total antioxidant capacity (TAOC), reflecting the contribution of plasma/serum water-soluble molecules to antioxidant capacity, including albumin, caeruloplasmin, transferrin, protein thiols, uric acid, ascorbic acid, and bilirubin, as well as some α-tocopherols, is an essential indicator of oxidative stress [24]. TAOC is thought to be involved in cognitive impairment in several groups, such as patients with Alzheimer’s disease (AD) and patients with early-onset first psychosis [25,26,27]. A previous study has found that TAOC levels in patients with paranoid schizophrenia are significantly lower than in healthy controls, suggesting a defect in the antioxidant system of schizophrenic patients [28]. Another study showed that TAOC levels were related to several cognitive deficits in schizophrenia patients, such as processing speed, attention/vigilance, and emotion management [29]. One possible mechanism is that, due to deficiencies in antioxidant defense, patients with schizophrenia exhibit more oxidative damage to lipids, proteins, and DNA in both central and peripheral tissues, thereby impairing cognitive-function-related neurons [30]. In addition, TAOC levels are currently a fairly important predictor of antipsychotic efficacy, suggesting that higher TAOC levels may predict better treatment responses to antipsychotics [31,32]. A recent study also suggests that the galantamine–memantine combination is promising as a novel antioxidant treatment for schizophrenia [33].

Given that TAOC levels might act as a biomarker of cognitive functions and are associated with oxidative stress, and the close relationship between cognitive function and age of onset in schizophrenia, in this study, our goal is to further investigate the inter-relationship between the age of onset, cognitive function, and TAOC levels in schizophrenia patients. In this study, cognitive function mainly represents five key domains: immediate memory, delayed memory, visuospatial/constructional, attention, and language. As far as we are concerned, this is the first study to examine the relationship between the age of onset, cognitive function, and TAOC levels in drug-naïve schizophrenia patients. Our hypotheses were that (1) there would be differences in cognitive performance and TAOC levels between EO and non-EO schizophrenia patients, and (2) there would be a positive correlation between TAOC levels and cognitive performance in the EO group. Therefore, the primary goals of our research were to investigate (1) whether clinical symptoms, cognitive functioning, and TAOC levels would differ between EO and non-EO schizophrenia patients, and (2) whether the age of onset would affect the association between TAOC levels and the cognitive functioning of schizophrenia patients.

## 2. Methods

### 2.1. Participants

In this study, we recruited 201 drug-naïve schizophrenia patients from a psychiatric hospital in China. Patients were diagnosed with schizophrenia based on the Structured Clinical Interview I for DSM-IV (SCID-I) criteria by two independent psychiatrists. Inclusion criteria were: (1) Han Chinese; (2) aged 14–55 years; (3) an illness duration of no more than 60 months; and (4) no history of antipsychotic or antidepressant treatment. Exclusion criteria were: (1) having major somatic diseases; (2) having a history of autoimmune diseases, allergies, hypertension, or diabetes; (3) being pregnant or breastfeeding; and (4) a current diagnosis of other psychiatric disorders, such as major depressive disorder or anxiety disorder. All subjects provided a comprehensive medical history as well as demographic data.

This study was approved by the Ethics Committee of Beijing Huilongguan Hospital (Ethics No. 2013-10). Each subject offered written informed consent before enrollment.

### 2.2. Defnition of EO and Non-EO Patients

Based on medical records, the age of first onset was determined as the age at which the participant first met DSM-IV criteria for schizophrenia. In this sample, the mean age of onset was 25.8 ± 9.6 years, with a range of 14 to 53 years. Depending on the age of onset, we classified these patients into two groups: <21 was the early-onset (EO) group and ≥21 was the non-early-onset (non-EO) group [6,34]. This age criterion effectively identified early-onset schizophrenia in previous studies [8].

### 2.3. Assessment of Psychiatric Symptoms and Cognitive Functions

Each patient completed a questionnaire about their sociodemographic features as well as medical and psychological status. Existing medical documents were used to gather further information. Participants whose data were lost or ambiguous underwent further interviews.

The Positive and Negative Syndrome Scale (PANSS) [35] was expert-based and was used by four trained psychiatrists to assess psychopathology. Repeated post-training assessments showed an inter-rater correlation coefficient of kappa = 0.84 for the PANSS total score.

Individual assessments of cognitive performance were conducted using the Repeatable Battery for the Assessment of Neuropsychological Status (RBANS, Form A) [36]. The 12 subtests that make up the RBANS were used to create a total score, as well as five age-adjusted index scores to measure five dimensions of cognitive functioning, including immediate memory, visuospatial/constructional, language, attention, and delayed memory. Trained psychiatrists scored the patient’s performance on the RBANS test.

### 2.4. Blood Sampling and TAOC Measurement

After an overnight fast, each subject had their venous blood sampled between 7:00 a.m. and 9:00 a.m. TAOC was then analyzed using established procedures [37]. The assay was fully described in our previous papers [31,37]. Briefly, ethylenediaminetetraacetic acid (EDTA) was used as an anticoagulant to obtain plasma. Two technicians used a commercially available kit (Nanjing Jiancheng Bioengineering Inc., Nanjing, China) to detect plasma TAOC. TAOC levels were tested as reductants from Fe^3+^ to Fe^2+^, which were then chelated by tripyridyl triazine (TPTZ) to produce a Fe^2+^-TPTZ complex, measured with a Multiskan microplate reader (FlowLabs, McLean, VA, USA). Blood TAOC levels were presented as units per milliliter of plasma (U/mL). The participants’ identities were disclosed by the code number kept by the lead investigator.

### 2.5. Statistical Analysis

First, we checked the normality of continuous variables using the Kolmogorov–Smirnov one-sample test. Levene’s Test for Equality of Variances was used to test for homoscedasticity. Based the Kolmogorov–Smirnov one-sample test results, we log-transformed the variables that did not follow the normal distribution, and then they all conformed to the normal distribution. In terms of outliers, we used box plots to identify outliers and values above 3 quartiles were considered outliers and excluded. Demographic, clinical, and cognitive characteristics were compared between the EO and non-EO groups using analysis of variance (ANOVA) for continuous variables and chi-square analysis for categorical variables. With Pearson correlation coefficients, associations between TAOC and demographic, clinical, and cognitive characteristics were assessed for the whole sample, and separately for EO and non-EO groups. Bonferroni correction was conducted to adjust for multiple testing.

To predict clinical features, a series of multiple linear regression analyses were performed. Initial requirements for conducting multiple regression analyses were met [38,39]: N = 201 > 100; the predictors can explain the dependent variables (e.g., R = 0.482, R^2^ = 0.233); the number of predictors was 6, 10 × 6 = 60 < N (200); and the Durbin–Watson coefficient was 1.94, suggesting that the residuals of the predictors were independent. In addition, the variance inflation factors (VIF) were used to assess multicollinearity, with values between 1.04 and 1.17, indicating little risk of multicollinearity. These analyses were performed with PANSS total or index scores, or RBANS total or index scores being the dependent variables to examine the effects of several variables, including age, gender, education, smoking status, BMI, and TAOC levels.

SPSS^®^, version 23.0 (IBM Corporation, Armonk, NY; USA) for Windows^®^ was used for all statistical analyses. Data are shown as mean ± SD. All *p* values were two-tailed and the significance level was set at 0.05.

## 3. Results

### 3.1. Demographic, Clinical and Cognitive Parameters in EO and Non-EO Patients

Table 1 presents the demographic, clinical, and cognitive variables of patients with EO and non-EO schizophrenia. The EO group included 90 patients (47 males and 43 females), while the non-EO group included 111 patients (60 males and 51 females). The EO group had a significantly lower age (*p* < 0.001), lower smoking rate (*p* < 0.001), lower education level (*p* = 0.007), lower BMI (*p* = 0.003), higher negative symptom subscore (*p* = 0.013, η^2^ = 0.01), lower visuospatial/constructional subscore (*p* = 0.003, η^2^ = 0.04), lower language subscore (*p* < 0.001, η^2^ = 0.095) and lower RBANS total score (*p* = 0.025, η^2^ = 0.03). Among all these significant variables, only differences in age, smoking rate, and language remained significant after Bonferroni correction (all corrected *p* < 0.05).

### 3.2. TAOC Levels in EO and Non-EO Schizophrenia Patients

**Figure 1:**
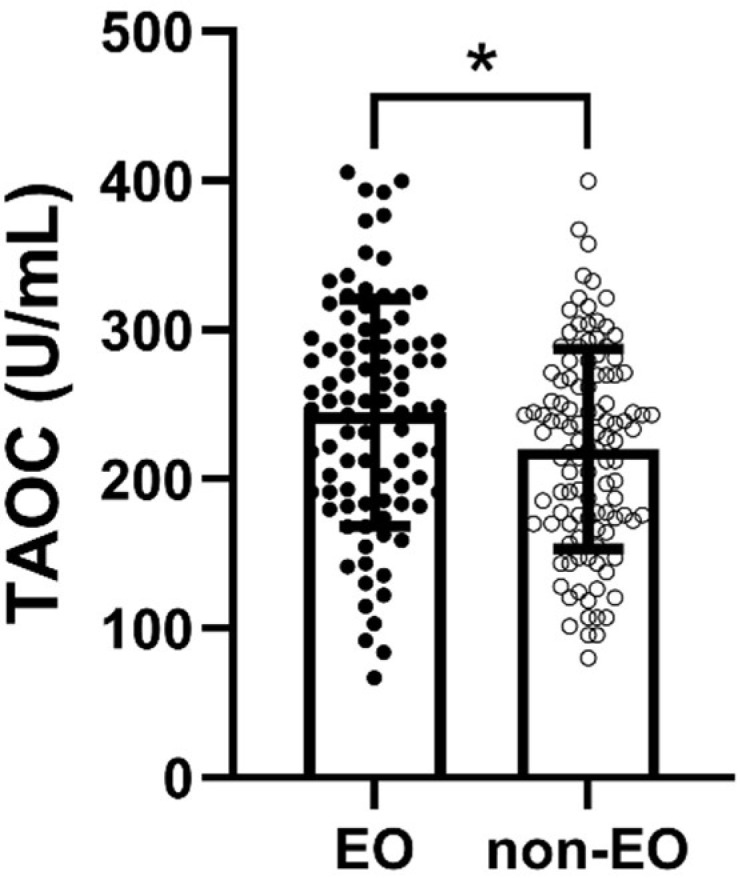
Comparison of TAOC levels between EO and non-EO groups. * refers to *p* < 0.05.

TAOC levels were considerably higher in the EO group than in the non-EO group (*p* = 0.016, η^2^ = 0.03) (Figure 1). Moreover, Pearson’s correlation analysis indicated a marginally significant correlation between TAOC levels and patients’ age of onset (*p* = 0.056) (Table 2). Nevertheless, there was no considerable relationship between age of onset and TAOC levels in either EO group or non-EO group (*p* > 0.05).

### 3.3. Association between TAOC Levels and Cognitive Function in EO and Non-EO Groups

The results of the correlation between TAOC and clinical or cognitive variables for the EO group, non-EO group, and all participants are shown in Table 2.

In the EO group, Pearson’s correlation analysis showed that TAOC levels were significantly correlated with attention (r = −0.217, *p* = 0.04). Nevertheless, this association retained its significance after the Bonferroni correction. Further multiple linear regressions showed no remarkable association between PANSS total and index scores, RBANS total and index scores, and TAOC levels. 

**Figure 2:**
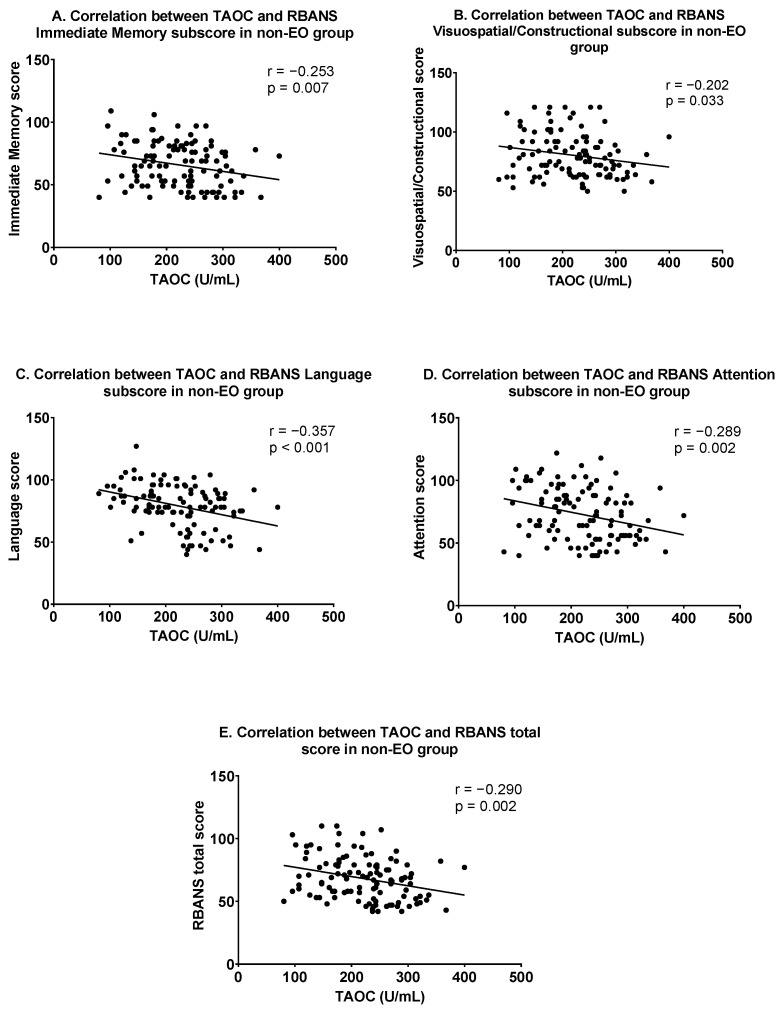
Correlations between TAOC and cognitive functions in the non-early-onset group. (**A**) Correlation between TAOC and RBANS Immediate Memory subscore; (**B**) Correlation between TAOC and RBANS Visuospatial/Constructional subscore; (**C**) Correlation between TAOC and RBANS Language subscore; (**D**) Correlation between TAOC and RBANS Attention subscore; (**E**) Correlation between TAOC and RBANS total score in non-EO group.

In the non-EO group, Pearson correlation analysis showed that TAOC levels were significantly related to the following variables: positive symptoms (r = −0.342, *p* < 0.001), general psychopathology (r = −0.265, *p* = 0.005), PANSS total score (r = −0. 346, *p* < 0.001), immediate memory (r = −0.253, *p* = 0.007), visuospatial/constructional (r = −0.202, *p* = 0.033), language (r = −0.357, *p* < 0.001), attention (r = −0.289, *p* = 0.002) and RBANS total scores (r = −0.290, *p* = 0.002). Figure 2 shows the correlation between TAOC and cognitive function in the non-EO group. After Bonferroni correction, only the associations between TAOC levels and positive symptoms, PANSS total score, language, attention, and RBANS total score continued to be significant (all corrected *p* < 0.05). Furthermore, using each clinical score as the dependent variable, multiple linear regression suggested that TAOC levels were independently correlated with the following: positive symptoms (β = −0.273, t = −2.498, *p* = 0.014), negative symptoms (β = −0. 320, t = −2.996, *p* = 0.004), general psychopathology (β = −0.236, t = −2.116, *p* = 0.037), and PANSS total scores (β = −0.373, t = −3.453, *p* = 0.001) and language (β = −0.192, t = −2.038, *p* = 0.044).

## 4. Discussion

There are three main findings in this research: (1) patients in the EO group had significantly more serious negative symptoms and more impaired cognitive performance than patients in the non-EO group; (2) TAOC levels were considerably higher in the EO group than in the non-EO group; and (3) after Bonferroni correction, TAOC levels were negatively correlated with positive symptoms, total PANSS scores, language, attention, and total RBANS scores only in the non-EO group. Our results suggest a difference in the relationship between TAOC levels and cognitive function in EO and non-EO patients, and age of onset may further moderate the relationship between TAOC levels and cognitive function in drug-naïve schizophrenia patients.

In the present study, it was found that EO patients had poorer cognitive functioning than non-EO patients, especially in the visuospatial/constructional and language domains. Our findings align with previous research that found people with EO schizophrenia had more severe impairments in various cognitive functions, such as verbal fluency and verbal memory [40,41]. Furthermore, our results suggest that EO patients have more serious negative symptoms than non-EO patients, which is also in line with the results of some earlier research [42,43]. However, some researchers have failed to find significant differences in cognition between EO and late-onset schizophrenia [44]. This inconsistency may be brought about by the small sample sizes, different measures of cognitive functioning, different stages of disease progression (e.g., first-episode versus chronic), different disease durations, and different treatment statuses (e.g., unmedicated versus medicated) in some previous research. In addition, different genetic backgrounds may play a vital role in the manifestation of the disease in EO patients and non-EO patients [45]. It can be assumed that EO may result from a monogenic disorder. In contrast, the manifestation of non-EO patients may be polygenic in nature, and may have different genetic causes for their phenotypic manifestations in different populations of subjects, resulting in different degrees of cognitive symptoms.

Several studies have shown a strong association between the age of onset and cognitive functioning in schizophrenia patients [46]. According to several reports, an earlier age of onset in schizophrenia patients is correlated with worse immediate memory, attention, social cognition, and verbal learning functions [18]. In addition, earlier age of onset is associated with abnormal functional connectivity between the striatum and other brain areas, supporting neurodevelopmental disruption in the early stages of schizophrenia [47]. It has also been shown that loss of cortical gray matter is noticed in the frontal lobes of patients with childhood-onset schizophrenia and that earlier onset predicts cognitive impairment due to frontal lobe damage [48,49]. This evidence suggests that earlier onset of schizophrenia may lead to more severe cognitive impairment by affecting brain structure and tissue damage. Furthermore, earlier onset is associated with more males, more negative symptoms, and higher doses of antipsychotic medication, which is often thought to indicate neurodevelopmental impairment [50]. In addition, more pronounced cognitive deficits in EO individuals may also be associated with higher rates of chromosomal abnormalities in schizophrenia and higher familial rates of schizophrenia spectrum disorders [51]. Age of onset has also been associated with the BDNF Val66Met gene polymorphism, which is related to poorer cognitive performance in schizophrenia [52,53]. Genetic and environmental risk factors may negatively affect the developing brain, resulting in an earlier age of onset and poorer cognitive performance [50]. More studies are needed to determine the exact mechanisms by which cognitive performance is worse in patients with EO schizophrenia than in non-EO patients.

We also found that TAOC levels were considerably different between the EO and non-EO groups, with EO patients having significantly higher TAOC levels than non-EO patients. Previous studies have shown considerable heterogeneity among studies regarding changes in antioxidant biomarker levels in patients with EO schizophrenia, even with opposite results [9]. These contradictory findings may be due to differences between subjects, different assessment and analysis methods, and clinical factors that may alter the levels of oxidative parameters (e.g., different levels of antipsychotic drug exposure). In the current research, higher TAOC levels were found in EO patients, possibly due to a compensatory effect on the acute rise in the pro-oxidant state, which is usually present in young schizophrenia patients [54]. Another possible reason is that oxidative stress levels in vivo naturally decrease with age [55], which may also partially explain the lower TAOC levels in non-EO patients.

It is worth noting that a negative correlation was found between TAOC levels and RBANS language, attention, and total score only in the non-EO group, which was out of our expectations. In non-EO patients, high TAOC may be compensatory, represent high levels of oxidative stress, and be associated with poorer cognitive function. Some previous reports have suggested that TAOC levels are correlated with cognitive dysfunction in schizophrenia patients in several domains, suggesting that oxidative stress might have an impact on the pathological process of cognitive dysfunction in schizophrenia patients [29]. However, some previous studies found no significant relationship between TAOC and cognitive impairment in schizophrenia, suggesting heterogeneity in the relationship between TAOC levels and cognitive function across studies [9]. Our findings indicated that the age of onset might have a moderating effect on the correlation between TAOC and cognitive function in schizophrenia patients. It was found that perineuronal networks (PNNs) mature in an experience-dependent way during late postnatal development, covering the prodromal/onset time of schizophrenia [56]. During adulthood, PNNs play a regulating role in neuronal properties, including sensitivity to oxidative stress [56]. Patients with EO schizophrenia often have PNNs that are not yet fully mature and have experienced damage due to the disease. In this case, the effect of oxidative stress indicators on cognitive function might become less significant. In addition, environmental factors such as long-term smoking and alcohol consumption accelerate telomere erosion as well as aging and affect the body’s oxidative stress levels [57]. Patients with relatively late-onset schizophrenia may experience long-term disruption of the oxidative stress system due to these environmental factors, which may also make the effects of oxidative stress on cognitive function more pronounced. In addition, the low sample size may be one of the possible reasons for the lack of association between TAOC levels and RBANS scores in the EO group. However, these are only our speculations and deserve further exploration in future studies.

Our study has the following limitations. Firstly, the cross-sectional design hindered causal reasoning about the association between cognitive impairments and TAOC levels in EO and non-EO schizophrenia patients. Future longitudinal studies are necessary to investigate the causal relationship between cognitive deficits and TAOC levels. Second, this study found considerable differences between the EO and non-EO groups in education, BMI, and smoking status. Although we controlled for these variables in the statistical analysis, matched subjects with similar demographic characteristics and socioeconomic status would be better in future studies. Third, this study primarily used TAOC as a representative marker of the antioxidant defense system. To gain more insight into the role of oxidative stress in cognitive impairment in schizophrenia, researchers need to systematically analyze all important synergistic oxidative stress markers in a larger cohort in future studies. Fourth, previous studies have shown that a higher oxidative stress index (OSI), defined as the ratio of total oxidants to total antioxidant status, reflects an unbalanced antioxidant status, which occurs in the early stages of schizophrenia and may play an important role in the negative symptoms of schizophrenia [58]. However, due to the limitations of blood sample analysis, our study did not include the OSI. Future studies are needed to explore the association between age of onset, OSI, and cognitive deficits in patients with schizophrenia. Fifth, we do not know the exact source of TAOC in our participants. It remains unclear whether the TAOC in the blood samples comes from the central nervous system and whether the TAOC levels in the peripheral system are the same as those in the cerebrospinal fluid. Sixth, we did not have a healthy control group in this study, which should be remedied in future studies. Finally, our study did not collect data on the duration of the prodromal period of patients in clinical practice; thus, we cannot use the prodromal period as a confounder in statistical analysis.

## 5. Conclusions

In conclusion, in the current study, we suggested that EO patients had significantly more serious negative symptoms and cognitive impairment than non-EO patients. We also found higher levels of TAOC in the EO group. In addition, a remarkable association between TAOC levels and patients’ clinical symptoms and cognitive function was found only in the non-EO group. TAOC levels may play a different role in cognitive deficits between EO and non-EO schizophrenia patients. This study provides clinical suggestions for treating non-EO schizophrenia, namely, that reducing patients’ oxidative stress levels may help improve their cognitive symptoms. In future studies, effective drugs could be developed to alleviate the negative effects of free radical products on cognitive function in schizophrenia. Therefore, randomized controlled trials are warranted with antioxidant therapy in schizophrenia. These findings may also help us better understand the relationship between oxidative stress and cognitive deficits in schizophrenia and the role of the age of onset in their relationship, providing more knowledge for future personalized treatment.

## Figures and Tables

**Table 1:** Demographics, clinical and cognitive variables in early-onset and non-early-onset patients with schizophrenia.

	EO (*n* = 90)	Non-EO (*n* = 111)	F or X^2^	df	*p* Value	Partial η^2^
	N (%)	N (%)				
Sex						
Male (%)	47 (52.22%)	60 (54.05%)	0.067	1	0.887	/
Female (%)	43 (47.78%)	51 (45.95%)				
Smoker (%)	10 (11.11%)	39 (35.14%)	13.992	1	<0.001	/
	M ± SD	M ± SD				
Education (y)	8.37 ± 2.33	9.78 ± 4.40	7.524	197	0.007	0.04
BMI (kg/m^2^)	20.31 ± 3.52	21.79 ± 3.25	8.990	186	0.003	0.05
Smoker (%)	10/90 (11.11%)	39/111 (35.14%)	13.992	1	<0.001	/
Age of onset (y)	17.79 ± 1.53	32.26 ± 8.48	255.259	200	<0.001	0.56
PANSS						
Positive symptoms	20.68 ± 5.66	21.40 ± 6.11	0.748	200	0.388	0.004
Negative symptoms	19.79 ± 6.94	17.44 ± 6.38	6.272	200	0.013	0.01
General psychopathology	34.48 ± 8.38	35.08 ± 8.75	0.246	200	0.621	0.001
Total Score	74.84 ± 14.66	73.64 ± 15.72	0.310	200	0.579	0.002
RBANS						
Immediate Memory	64.12 ± 14.99	66.00 ± 17.59	0.647	200	0.422	0.003
Visuospatial/Constructional	73.38 ± 13.81	80.39 ± 18.38	9.015	200	0.003	0.04
Language	67.91 ± 18.21	79.33 ± 17.15	20.927	200	<0.001	0.095
Attention	76.34 ± 18.30	72.86 ± 21.03	1.538	200	0.216	0.01
Delayed Memory	66.41 ± 19.94	70.86 ± 20.14	2.453	200	0.119	0.01
Total Score	63.31 ± 13.34	68.29 ± 17.05	5.134	200	0.025	0.03

Note: M = mean; % = percentage of sample who reported; SD = standard deviation; EO = early-onset; Non-EO = non-early-onset; BMI = body mass index; PANSS = Positive and Negative Syndrome Scale; RBANS = Repeatable Battery for the Assessment of Neuropsychological Status; TAOC = Total Antioxidant Capacity.

**Table 2:** Correlations between TAOC and clinical and cognitive variables in early-onset, non-early-onset and all patients with schizophrenia.

	EO (*n* = 90)	Non-EO (*n* = 111)	All Participants (*n* = 201)
	TAOC	TAOC	TAOC
Age of onset	0.121 (0.256)	−0.039 (0.685)	−0.135 (0.056)
BMI (kg/m^2^)	−0.176 (0.109)	−0.180 (0.068)	−0.212 (0.003)
PANSS			
Positive symptoms	−0.180 (0.089)	−0.342 (0.000)	−0.273 (0.000)
Negative symptoms	0.038 (0.719)	−0.158 (0.096)	−0.029 (0.681)
General psychopathology	0.016 (0.883)	−0.265 (0.005)	−0.138 (0.050)
Total Score	−0.042 (0.693)	−0.346 (0.000)	−0.196 (0.005)
RBANS			
Immediate Memory	−0.112 (0.295)	−0.253 (0.007)	−0.196 (0.005)
Visuospatial/Constructional	−0.064 (0.548)	−0.202 (0.033)	−0.173 (0.014)
Language	−0.134 (0.208)	−0.357 (0.000)	−0.284 (0.000)
Attention	−0.217 (0.040)	−0.289 (0.002)	−0.236 (0.001)
Delayed Memory	−0.105 (0.323)	−0.163 (0.086)	−0.151 (0.032)
Total Score	−0.172 (0.104)	−0.290 (0.002)	−0.258 (0.000)

Note: TAOC = Total Antioxidant Capacity; EO = early-onset; Non-EO = non-early-onset; BMI = body mass index; PANSS = Positive and Negative Syndrome Scale; RBANS = Repeatable Battery for the Assessment of Neuropsychological Status; Pearson’s correlation coefficient was used to assess the correlations between TAOC levels and the other variables mentioned above, shown as r (*p*-value).

## Data Availability

All of the data is contained within the article.
